# An Estimation of Future Heat- and Cold-Associated Mortality in the Largest 15 Cities in Germany

**DOI:** 10.3238/arztebl.m2025.0180

**Published:** 2025-12-12

**Authors:** Susanne Breitner-Busch, Pierre Masselot, Veronika Huber, Antonio Gasparrini, Alexandra Schneider

**Affiliations:** Institut für Epidemiologie, Helmholtz Zentrum München – Deutsches Forschungszentrum für Gesundheit und Umwelt (GmbH), Neuherberg, Germany; Lehrstuhl für Epidemiologie, Institut für Medizinische Informations-verarbeitung, Biometrie und Epidemiologie (IBE), Medizinische Fakultät, Ludwig-Maximilians-Universität (LMU) München, Germany; Environment and Health Modelling (EHM) Lab, Department of Public Health, Environment and Society, London School of Hygiene & Tropical Medicine, London, United Kingdom; Doñana Biological Station (EBD), Spanish National Research Council (CSIC), Sevilla, Spain; Environment and Health Modelling (EHM) Lab, Department of Public Health, Environment and Society, London School of Hygiene & Tropical Medicine, London, United Kingdom; Institut für Epidemiologie, Helmholtz Zentrum München – Deutsches Forschungszentrum für Gesundheit und Umwelt (GmbH), Neuherberg, Germany

The potential effects of climate change on public health have gained increasing importance in recent years, particularly with regard to temperature-associated mortality. While the number of cold-associated deaths in Europe is currently significantly higher than the number of heat-associated deaths (1), it remains unclear how this balance could shift in the future as temperatures continue to rise as a result ofclimate change.

A study recently published in Nature Medicine (2) investigated the potential future effects of climate change on heat- and cold-associated mortality in 854 European cities, including 117 in Germany. While the primary focus of the study by Masselot et al. (2) was on the European perspective, our article focuses specifically on the results for Germany and, more particularly, on the 15 largest German cities. To this end, we examined various climatic, demographic, and adaptation scenarios in order to comprehensively assess potential health effects.

## Methods

This study is based on multiple data sources, including an analysis of the associations between temperature and mortality (3) that quantifies the effect of temperature on mortality in urban areas. In addition, daily temperature projections for the period 2015–2099 from global climate models combining warming and socioeconomic scenarios of varying severity were used (4). The following scenarios were considered:

- SSP1–2.6 (a sustainable world characterized by limited global warming)
- SSP2–4.5 (slow progress toward sustainability)
- SSP3–7.0 (a world characterized by strong regional rivalries and the potential for substantial global warming).

These projections were calibrated using historical air temperature series (2, 5). Demographic data were also used to estimate future population sizes and age distributions.

Based on the various projection data and the temperature–mortality relationships, we determined the future number of temperature-associated deaths and mortality rates using standard methods (2, 3). In order to isolate the effects of climate change on mortality, two sub-scenarios were compared:

- A “complete” scenario that takes into consideration not only temperature changes but also demographic shifts
- A “demographic changes only” scenario, in which the temperature distribution remained consistent with that observed during the 2000–2014 period.

This approach enabled a more differentiated understanding of the effects of temperature changes and demographic trends on temperature-associated mortality. The results presented below take into account both a baseline scenario without population-level heat adaptation and a scenario involving a 10% adaptation *(Figure)*.

## Results

We identified an increase in the net effect (increasing heat effects plus decreasing cold effects) on temperature-associated excess mortality by 2099 in almost all of the 15 largest German cities across the three SSP scenarios considered. This net effect is all the greater the more pessimistic the scenario (SSP2–4.5 and SSP3–7.0) and tends to be more pronounced the further south in Germany the city is located (Figure).

By the middle of the century (2050–2054), the temperature-associated excess mortality rates per 100 000 person-years for the net effects—assuming the most pessimistic warming scenario (SSP3–7.0)—are 5.5 (95% confidence interval [–11.3; 37.3]) for the 15 largest cities and 6.5 [–11.0; 39.4]) for all 117 German cities. By the end of the century (2095–2099), these values rise to 34.0 [–8.2; 96.4] and 36.9 [–8.1; 103.8], respectively. Expressed as cumulative absolute deaths totaled over all years, this corresponds to 3571 [–33 399; 36 322] additional temperature-associated deaths for the 15 largest cities by the middle of the century (2050–2054) and 80 333 [–38 589; 263 899] by the end of the century (2095–2099), representing an increase in deaths of approximately 1%. For all 117 German cities, the corresponding estimates are 10 536 [–76 062; 86 957] and 190 330 [–90 386; 610 063] additional temperature-associated deaths, respectively. If one compares the temperature-associated excess mortality rates in relation to different future warming levels (1.5°C and 3°C) based on the SSP 3–7.0 scenario, a relatively homogeneous picture across cities emerges, with the smallest effects seen in Hamburg (Table).

## Discussion

Our results indicate substantial effects of climate change on temperature-associated mortality in Germany. While cold-associated mortality is expected to decline, heat-associated mortality is projected to increase. This will lead to a net increase in temperature-associated mortality, particularly in the second half of the century. In addition to demographic challenges and a shift in the mortality balance, the study also highlights the need for regional measures. Since the health risks associated with climate change differ across Germany, tailored strategies that take into account local geography, socioeconomic factors, and public health infrastructure will be crucial for effective adaptation to climate change. In summary, the study underscores the health risks that climate change poses for German cities. While milder winters may reduce cold-associated mortality, the projected rise in heat-associated deaths calls for urgent measures. The implementation of effective adaptation and mitigation strategies is crucial for protecting public health and ensuring that urban populations are able to withstand climate change.

## Figures and Tables

**Figure:**
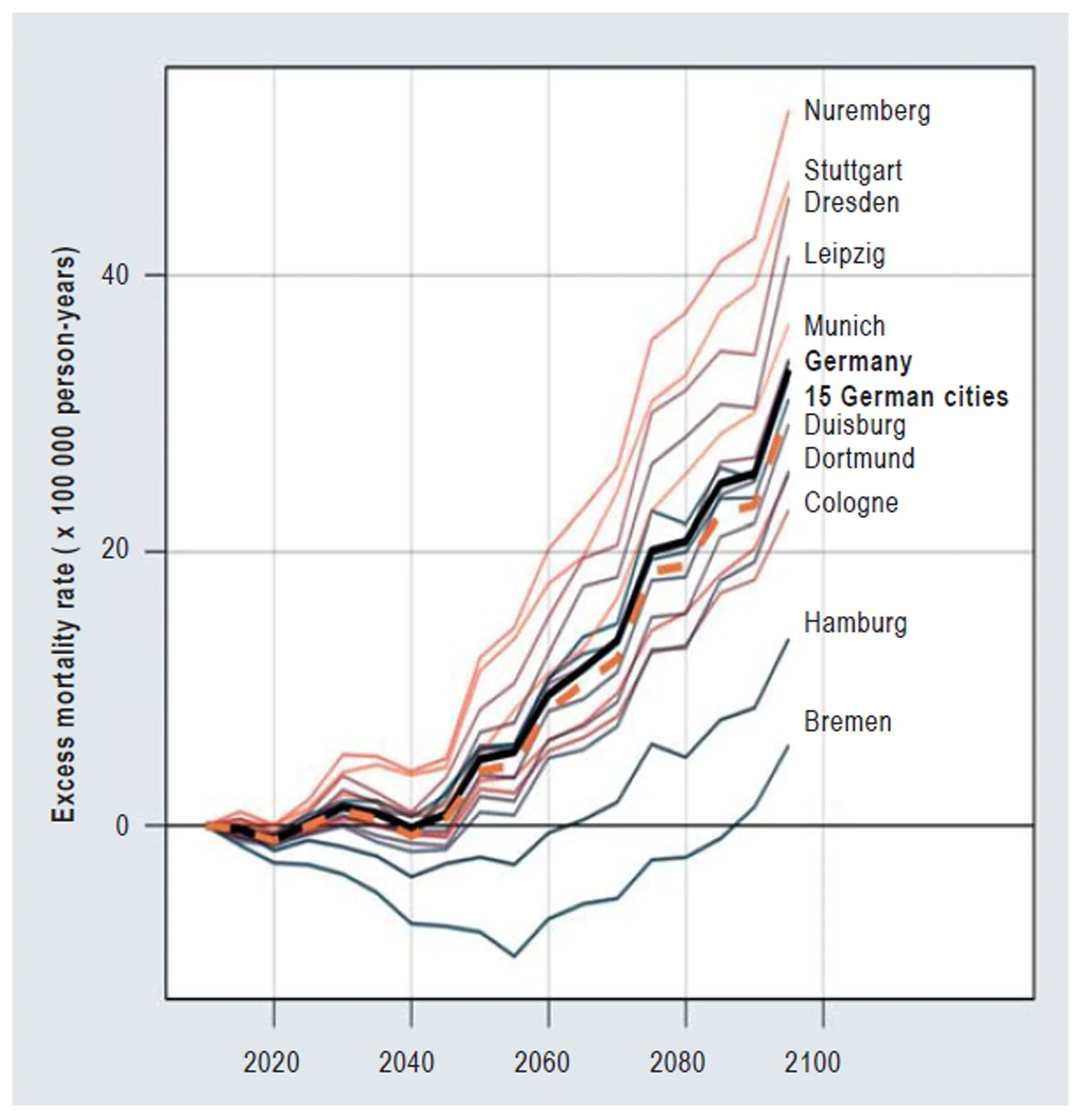
**Projection of the net changes in the temperature-associated excess mortality rate** from 2015 to 2099 under the SSP3–7.0 warming scenario, assuming that population adaptation to heat (a 10% reduction in mortality risk) occurs. The projections for the 15 largest German cities (individually and collectively, dashed line) as well as a projection for Germany that includes 117 German cities (thick black line), are shown.

**Table:** Temperature-associated excess mortality rates per 100 000 population for cold, heat, and net effects at different levels of warming (1.5°C and 3°C)*

	1.5°C			3°C		
	Cold effect	Heat effect	Net effect	Cold effect	Heat effect	Net effect
**Berlin**	–6.0 [–19.0; 2.4]	6.3 [–4.3; 26.3]	0.3 [–12.8; 19.6]	–23.0 [–40.6; –9.4]	33.6 [4.8; 80.7]	10.6 [–16.5; 52.6]
**Frankfurt am Main**	–4.8 [–13.9; 0.4]	5.1 [–3.0; 21.2]	0.2 [–9.8; 13.7]	–19.0 [–32.1; –9.4]	25.8 [2.2; 66.3]	6.8 [–15.4; 44.9]
**Hamburg**	–5.1 [–18.1; 4.1]	3.4 [–4.3; 17.8]	–1.7 [–14.8; 11.4]	–22.0 [–39.4; –8.9]	20.8 [1.3; 57.2]	–1.2 [–21.7; 32.2]
**Cologne**	–4.7 [–15.4; 1.7]	4.6 [–3.5; 19.0]	–0.2 [–12.9; 13.1]	–18.6 [–33.7; –7.6]	23.9 [3.1; 62.1]	5.3 [–17.8; 39.9]
**Leipzig**	–7.2 [–23.6; 3.6]	8.5 [–3.3; 30.3]	1.3 [–16.3; 23.0]	–28.2 [–49.1; –12.1]	41.1 [7.4; 92.8]	12.9 [–19.5; 59.7]
**Munich**	–5.4 [–14.3; 0.0]	7.4 [–1.0; 25.6]	1.9 [–7.3; 17.9]	–19.9 [–33.0; –10.3]	33.6 [5.0; 89.3]	13.7 [–12.0; 67.9]
**15 Largest cities**	–5.7 [–17.5; 1.5]	6.0 [–2.7; 23.3]	0.3 [–12.2; 15.6]	–22.5 [–38.5; –10.6]	31.1 [5.9; 74.2]	8.6 [–15.8; 46.5]
**Germany**	–5.7 [–17.4; 1.4]	6.3 [–3.0; 24.1]	0.7 [–12.5; 16.4]	–22.7 [–38.9; –10.6]	32.6 [5.8; 77.6]	9.9 [–16.0; 50.8]

* Based on the SSP 3–7.0 scenario, assuming that no population adaptation to heat occurs. The effects for the largest German cities (as examples and collectively), as well as the effects for Germany as a whole, based on 117 German cities, are shown.
